# A qualitative study of barriers to employment experienced by people living with HIV in Toronto and Ottawa

**DOI:** 10.1186/s12939-020-01356-4

**Published:** 2021-01-14

**Authors:** Melissa Perri, Amy-Craig Neil, Mark Gaspar, Charlotte Hunter, Claire Kendall, Ower Alexander, Andrew D. Pinto

**Affiliations:** 1 Upstream Lab, MAP/Centre for Urban Health Solutions, Li Ka Shing Knowledge Institute, Unity Health Toronto, 30 Bond St, Toronto, Ontario M5B 1W8 Canada; 2 Dalla Lana School of Public Health, University of Toronto, 155 College St, Toronto, Ontario M5T 3M7 Canada; 3 Casey House, 119 Isabella St, Toronto, Ontario M4Y 1P2 Canada; 4 Department of Family and Community Medicine, St. Michael’s Hospital, 500 University Ave, Toronto, Ontario M5G 1V7 Canada; 5 Department of Family Medicine, University of Ottawa, 600 Peter Morand Crescent Suite 201, Ottawa, Ontario K1G 5Z3 Canada; 6 C.T. Lamont Primary Health Care Research Centre, Bruyère Research Institute, 85 Primrose Ave, Ottawa, Ontario K1R 6M1 Canada; 7 Institut du Savoir Montfort, Montfort Hospital, 713 Montreal Rd, Ottawa, Ontario K1K OT2 Canada; 8 Ottawa Hospital Research Institute, 501 Smyth Box 511, Ottawa, Ontario K1H 8L6 Canada; 9 Li Ka Shing Knowledge Institute, 209 Victoria St, Toronto, Ontario M5B 1T8 Canada; 10 University of Toronto Practice-Based Research Network, 500 University Avenue, Toronto, Ontario K1H 8L6 Canada

**Keywords:** HIV, Employment, Barriers, Social determinants of health

## Abstract

**Background:**

Effective treatment has extended the life expectancy and reduced disability in people living with HIV (PLWH). However, previous research has found 45–65% of working-age PLWH were unemployed compared to 5–10% in the general public of North America and Europe. We examined the barriers to gaining employment among PLWH.

**Methods:**

Thirty-five in-depth interviews were conducted in person or over the phone with PLWH living in Toronto or Ottawa. This included PLWH who were unemployed but actively seeking employment, as well as PLWH who had successfully gained employment through an agency that specifically supported PLWH funded by the AIDS Committee of Toronto. Interviews were conducted between February 2019 and March 2020. All interviews were audio-recorded, transcribed and analyzed using thematic analysis.

**Results:**

The majority of participants were between the ages of 40–55 and identified as male. Participants shared many common barriers when describing their attempts to attain or maintain employment. Although varying in employment status at the time of the study, consistent barriers included experiencing HIV stigma in workplaces, challenges overcoming mental health illnesses, and difficulties in navigating social assistance and unemployment insurance programs when pursuing a return to work.

**Conclusions:**

PLWH face significant barriers when attempting to engage with employment opportunities. Health providers and organizations can do more to support campaigns to end HIV stigma, to support individuals in pursuing employment, and to advocate for policy change that supports reentry into the workforce for PLHA.

**Supplementary Information:**

The online version contains supplementary material available at 10.1186/s12939-020-01356-4.

## Background

Over 68,000 people are living with HIV in Canada [1]. In the early phase of the HIV epidemic, individuals experienced significant morbidity and mortality [2]. However, effective and increasingly tolerable antiretroviral treatment has shifted HIV from a terminal to a chronic condition [3, 4]. As such, the quality of life of PLWH has improved, with increases in function allowing them to more easily engage in everyday activities [3, 5].

Employment is a fundamental determinant of health and well-being, influencing income, access to shelter, healthcare, social capital and food security [6–12]. Studies among PLWH specifically have found that employment directly contributes to improved physical and mental health; increased income, self-esteem, and social interactions; and decreased reliance on disability programs [13–15]. Unsurprisingly, many unemployed PLWH express an interest in returning to the workforce [16].

Despite this increased functional capacity and personal interest in contributing to the workforce, unemployment rates remain high among PLWH [6]. Studies from Canada, United States and France estimate that 45–65% of working-age PLWH were unemployed, compared to 5–10% in the general public of these countries respectively [17–20]. This disparity is a likely a result of several barriers to sustaining employment still faced by PLWH including unpredictable periodic disability [21], persistent physical limitations, gaps in employment history and difficulty in navigating social assistance plans [22, 23]. Similar findings have been documented in the United States and Australia, outlining barriers to employment including the development of social assistance plans, age, co-morbidities, and experiences of mental health illness [24–26].

Reviews of social policies that impact PLWH in Canada have found that employment and income policies can be poorly coordinated and unduly penalizing. For example, social access to prescription drug coverage may be lost if PLWH enter into employment [27]. To address these concerns, several interventions specifically targeted to helping PLWH enter the workforce exist, including the AIDS Committee of Toronto’s (ACT) Employment ACTion (EA) program or the Black Coalition for AIDS Prevention’s Kazi Employment Program.

We sought to understand the barriers faced by PLWH in gaining or maintaining employment. This study uniquely assesses common barriers to employment among PLWH who are not employed but actively seeking to re-enter the workforce and those who had previously engaged with EA, an employment specific service.

## Methods

### Design

This qualitative, community-based research study aimed at understanding what barriers exist to employment among PLWH residing in urban centres in Ontario.. The research team included ACT as a community partner.

### Setting

This study was conducted in Toronto and Ottawa, Canada. Toronto was chosen as it has the highest number of PLWH in Ontario, and is home to the EA program [28]. Ottawa, a similarly populated region, has been documented as having the second highest rates of HIV in Ontario, and therefore was chosen as an alternative urban setting [28]. Our study team has expertise in the social determinants of health, HIV clinical care and epidemiology. We collaborated with the AIDS Committee of Toronto (ACT), an AIDS Service Organization (ASO) that has supported PLWH to engage in employment through their EA program since 1999 [29]. EA aims to assist PLWH who reside in Toronto in gaining effective employment [29]. Through the use of interdisciplinary services, which are tailored to PLWH, service providers associated with EA assist community members in increasing skills relating to attaining employment (i.e. vocational training). The study was reviewed and approved by the Unity Health Toronto, Ottawa Health Science Network and Bruyère Research Institute Ethics Boards.

### Sampling and data collection

We used purposive sampling to recruit participants. Participants had to be working-age (18–64 years old), a PLWH who was unemployed but actively seeking employment, or those who had successfully gained employment and held it for at least 13 weeks through working with EA. To recruit those seeking employment, posters and emails were circulated by HIV clinics, primary care clinics serving large numbers of PLWH and at AIDS Service Organizations in both Toronto and Ottawa between February 2019 and March 2020. To recruit individuals that had successfully been placed in employment through EA, we circulated an email to all previous clients, and posted posters (Additional file 1: Appendix 1A & 1B) in EA offices.

We used semi-structured interviews, which were conducted by one research coordinator based at the Upstream Lab (ACN), who has significant experience in conducting qualitative-based interviews. All interviews took place either in person or over the phone. The interview guides (Additional file 2: Appendix 2A, 2B & 2C) were developed with an interdisciplinary team of stakeholders (i.e. clinicians who focus on HIV, HIV-specific service agencies and individuals with lived experiences of HIV). Prior to the interview, participants completed the informed consent process and a demographic survey. During both sets of interviews, participants were probed about barriers faced in attaining and maintaining employment and the relationship between their health and employment status.

Data were determined to reach theoretical saturation when no new themes or viewpoints were emerging from the interviews [30].

### Analysis

All interviews were audio-recorded and transcribed by a professional transcription company. We used iterative thematic analysis to code the transcripts in NVivo 11 [31]. First, five team members (ACN, MP, AP, CH and MG) independently reviewed manuscripts from each participant group, with the aim to inductively analyze and determine naturally apparent codes. Each member met and discussed apparent themes and agreed on the coding categories that were incorporated into the final coding framework. The remaining transcripts were then analyzed by two members of the study team (ACN and MP). Once completed, the team members convened to review overall themes and confirm the findings. Interview data among the different groups that similarly discussed barriers were consolidated when reporting the study findings. Preliminary long-form results were shared four times with the entire team, which provided additional input on interpretation of data to finalize the results below.

## Results

We conducted a total of 35 interviews, which lasted between 15 min and 1 h in Toronto (*n* = 17 unemployed; *n* = 12 who had gained employment through EA) and Ottawa (*n* = 6 unemployed). Employed individuals were only interviewed in Toronto as Ottawa did not have a program which was similar to EA. Among unemployed respondents interested in the study, 12 individuals in addition to the interviewed sample were ineligible, and 8 were unable to be contacted. In the EA group, one initially interested respondent declined and one was unable to be contacted.

Unemployed participants from Toronto were between the ages of 34 and 62, with a median age of 42 (Standard Deviation (SD) = 8.6) (Table 1). The majority identified as male (*n* = 12, 71%) and had completed a college or university degree (*n* = 10, 59%). Unemployed participants from Ottawa ranged from 37 to 58 years old, with a median age of 52 (SD = 6.7). The majority identified as male (*n* = 4, 67%) and were born in Canada (*n* = 5, 83%). Participants employed through EA, had a median age of 47 years old (SD = 10.0), with ages ranging between 27 and 64, the majority were male (*n* = 10, 83%) and completed a college or university degree (*n* = 11, 92%). None of the participants in this group were born in Canada (*n* = 12, 100%).

**Table 1:** Demographic characteristics of participants

	Toronto (Unemployed) *n* = 17	Ottawa (Unemployed) *n* = 6	Toronto (EA) *n* = 12	Total *N* = 35
Age				
26–39	5 (29%)	1 (17%)	4 (33%)	10 (29%)
40–55	9 (53%)	3 (50%)	7 (58%)	19 (54%)
56–64	3 (18%)	2 (33%)	1 (8%)	6 (17%)
Gender				
Male	12 (71%)	4 (67%)	10 (83%)	26 (74%)
Female	5 (29%)	2 (33%)	2 (17%)	9 (26%)
Highest Level of Education				
Did not complete high school	1 (6%)	1 (17%)	0 (0%)	2 (6%)
Completed high school	3 (18%)	2 (33%)	1 (8%)	6 (17%)
Completed some college or university	4 (24%)	2 (33%)	0 (0%)	6 (17%)
Completed college or university	9 (53%)	1 (17%)	11 (92%)	21 (60%)
First Language				
English	9 (53%)	6 (100%)	5 (42%)	20 (57%)
Other	8 (47%)	0 (0%)	7 (58%)	15 (43%)
Born In Canada				
Yes	6 (35%)	5 (83%)	0 (0%)	11 (21%)
No	11 (65%)	1 (17%)	12 (100%)	24 (69%)

Both the EA and unemployment group discussed similar and consistent barriers which were faced when attempting to engage with employment: HIV-related stigma, mental health concerns, and the design of social assistance and unemployment insurance programs.

### HIV-related stigma and discrimination are major concerns when seeking employment

HIV-related stigma was described as the greatest barrier to gaining employment. Participants attempting to start or seek new employment opportunities feared disclosure to employers and co-workers of their HIV status due to the anticipated stigma they might face. Individuals expressed worry that employers would find out about their HIV status through accessing drug benefits, leading to a termination of contracts. As one participant noted, *“I saw people going back into the workforce and then going right back on the social assistance because of employers who once they found out about their [HIV] status they would find a way to arrange to, that they would get fired or something”* (Male, 52, unemployed, Ottawa). Another commented, “*I was afraid people would know about my status and also if they know about my status if they’d hire me because of the insurance and all that* (Female, 51, EA, Toronto) and another “*I feel that there is a big stigma attached to people living with HIV… it’s just like the stigma and feeling attacked and stigmatized and discriminated against at work*” (Male, 27, EA, Toronto).

Participants were also fearful about experiencing discrimination and stigma from co-workers or undergoing repercussions such as hate crimes, which had impacted overall health and well-being. One participant outlined the significant toll HIV-related stigma had on well-being, *“It’s not the virus that kills people, it’s the stigma. When people are running away from you, you get thinking. When you are thinking, depression will set in. When you are depressed, before you know, your immune starts going down. You start losing weight, you are not doing exercise, you start staying away from people. You are isolated. So before the person know, the person is dead already”* (Female, 36, unemployed, Toronto). Another described how widespread it can be, “*there is a huge stigma in every field and that stigma hurts people*” (Male, 30, EA, Toronto).

Stigma was experienced both in and outside of the work environment. Consequently, some participants reported isolating themselves from employment as well as other social activities, educational opportunities and relationships. One participant spoke about how the harmful discrimination that PLWH face impact their ability to meaningfully engage in all aspects of life, “*If you got HIV and you’re gay and you’re a drug addict or you’re a prostitute or you’re undesirable like a member of society”* (Male, 58, unemployed, Ottawa). Another mentioned, *“I think in the long run everybody going to realize okay, let’s take down the stigma walls and just everybody you know survive and live you know”* (Female, 56, unemployed, Ottawa).

### Mental health was a significant barrier to PLWH seekingemployment

Although occurrences of mental illness also present barriers to the general public in attaining and sustaining employment [32], participants described a link between HIV-related status and diagnosis to their development of mental health conditions, such as anxiety and depression. When asked about how these conditions acted as barriers to employment, one participant outlined, *“But mostly the anxiety disorder, like being around people, so that’s like riding the TTC to work, working in general with people around you, job interviews”* (Male, 39, unemployed, Toronto).

Many patients described a sense of anxiety in thinking about pre-employment duties such as interviews or training, due to their age, time away from work or limited confidence. One participant described, “*So it makes me extremely anxious. I figure I will have to go through some kind of employment training thing, though, because I have nothing to offer really”* (Male, 39, unemployed, Toronto).

Participants also noted an association between stigma related to unemployment itself and mental health. Occurrences of mental illness were more frequent and severe when participants had experienced both unemployment and HIV-related stigma. As one individual noted, “*I’ve identified the fact that I’m really hard on myself because of my HIV hence it’s interfered with my gaining, my gaining employment* (Male, 52, unemployed, Ottawa). While another participant said, “*I have social anxiety and anxiety [generalized]. I don’t have many friends so I try to keep it to myself. I don’t tell the friends that I have, that I am not employed”* (Male, 61, unemployed, Toronto). Another mentioned, “*the huge stigma that I was talking about and the side effects of that stigma, yeah, because it was affecting my psychology and when you feel bad you will…I felt anxiety.”* (Male, 30, EA, Toronto).

### Navigating social assistance and unemployment insurance programs significantly impacted employment attainment among PLWH

A theme arising among both the unemployed and EA participants related to social support under the provincial disability program (Ontario Disability Support Program, ODSP). Receiving ODSP meant individuals qualified for prescription medication benefits, which they often feared would be lost if they were to gain employment. Many worried that if they then subsequently became unemployed again they would be left with no income and no drug benefits. Participants also expressed concern that their income from employment would be less than that obtained through ODSP. One participant stated, *“When you start work your rent goes up because where I’m living it’s subsidized housing so when I start to work it’s like, it got, it goes up to $1,000.00 and if I work for like $2,000.00 a month that’s huge I think my OSDP will be cut and then I am not sure I am going to get drug [benefits]”* (Male, 34, unemployed, Toronto). Another noted, “*I’m still worried because when you start working and based on your income they deduct the amount that you get… I was fearful that I would have lost benefits but I’ve still lost; right, because I’m only getting like drug coverage and transportation but so the majority of funding has been cut*.” (Female, 47, EA, Toronto).

The confluence of income insecurity and the high cost of medication resulted in many participants deciding to remain on ODSP rather than attaining employment. For many, staying out of the workforce was explicitly to ensure continual access to their medications. As one participant explained, *“At the end of the day when I look at it, it’s like I might actually find myself working throughout the days [of] a week but actually getting less than I can get right now. I don’t have, how could I get my drugs and how could I pay for my rent? It’s sort of like keeping people in this you know position”* (Male, 61, unemployed, Toronto). Another described, “*it would be ridiculous for me even to find full-time job in the kitchen. I will get for working all month I will get maybe $400.00, $500.00 extra and question is like would I not lose benefits after some times like who knows or, and I will end up being sick*” (Male, 48, EA, Toronto).

## Discussion

We identified a number of key barriers PLWH face to gaining employment. Although some barriers are not specific to living with HIV [33–35], we did find unique challenges among PLWH, namely ongoing HIV stigma in the workplace and the influence of social assistance programs as barriers to employment. Many participants described how barriers reinforce one another, such as how experiencing HIV stigma reinforces mental illness, or how difficulty in obtaining drug coverage while struggling to make a sufficient income was a major challenge in pursuing employment.

This paper adds to the body of knowledge surrounding the impacts and pervasiveness of stigma among PLWH [36–38]. Even among PLWH who had utilized a tailored employment program, barriers to employment were consistent. Experiences of stigma impact multiple areas of life including access to income, employment and health care [38, 39] and stigma contributes to health and social inequities among PLWH [36, 38, 39]. Our study is consistent with studies in other countries that have documented how HIV-related stigma prevent PLWH from meaningfully engaging with employment opportunities. Liu and colleagues [40] found that when engaging with PLWH, employers’ willingness to interview was dependent on fear of contagion and preconceived perceptions on PLWH capacity. Similarly, Martin and colleagues [41] who conducted a survey among PLWH, found that factors such as fear of discrimination, gaps in work-related experience and lack of vocational skills impacted PLWHs’ perceptions surrounding attaining employment [16]. Barrington and colleagues [42] found that HIV status and HIV-related stigma resulted in job loss among PLWH. This same study documented that some PLWH were tested for HIV without consent and subsequently let go from places of employment [42]. Additional studies have also determined the link between mental health disorders among PLWH and challenges in attaining employment [16, 43].

Our study highlights how the structure of social assistance programs – where medication benefits are tied to participation in of a program – can be a barrier to pursuing employment. The complexities faced by these groups when attempting to navigate drug coverage while attaining employment has resulted in many PLWH choosing to solely remain on social assistance programs. This finding is important in the discussion surrounding the need for universal drug coverage, particularly due to the incredibly high costs of HIV medication [2–4, 44]. There is a need to reform social assistance programs to automatically allow previous social assistance recipients to maintain medication benefits and to allow them to revert back to social assistance if needed. It is also important that there is transparency about these regulations and that this process is easy to navigate. This could create confidence among PLWH in engaging with employment opportunities.

A strength of this study was the consistency of responses across respondents. A limitation of the study was that all participants were likely connected to care, as they were recruited through ASOs or health care organizations. All interviews were conducted in English, and we did not collect data on race or ethnicity. We may not have captured the barriers faced by the most socially marginalized or isolated individuals. Another limitation is that all of the participants in the EA group were born outside of Canada however this may reflect the that people from endemic countries have higher incidences of HIV than the general population of Ontario [45].

Our findings shed light on the ongoing reality faced by PLWH in navigating health and social systems. The stigma experienced by PLWH creates immense barriers in attaining employment and maintaining overall well-being. Moving forward, institutions both inside and outside of the employment sector must engage in anti-stigma work [46], and provide support to those who have experienced HIV-related stigma. In addition to implementing anti-stigma frameworks, employment programs must begin to integrate mental health supports. Addressing structural stigmatization must also occur within the health care system. Physicians should be mindful of the HIV-associated stigma and mental health concerns their patients may face within the employment sector when having discussions about re-entering the workforce. Another focus should be on reforming social assistance and disability support programs to support re-entry into the workforce for PLWH who hope to do so. One aspect is examining how drug coverage intersects with employment among PLWH, and ensuring that medication access continues regardless of employment status. Other aspects include low barriers to re-training and education, tailored advice and connections to employers, and easy re-entry to social assistance and disability support programs if a job is lost.

## Conclusion

This qualitative study aimed at understanding key barriers faced by PLWH when attempting to attain employment. The consistently apparent link between HIV stigma and barriers faced when attempting to engage in employment that were described by our participants, provides an objective for future interventions to address. Developing tailored interventions with and for PLWH, with a strong emphasis on anti-stigma and mental health supports, and support when exiting and re-entering social assistance and disability support programs, are essential in minimizing current health and social disparities faced by these groups across North America.

## Supplementary Information

**Additional file 1.** Recruitment Posters for PLWHs Employed and Unemployed.

**Additional file 2: Appendix 2A:** Interview guide for interviews with PHAs unemployed but interested in re-entering work. **Appendix 2B**: Revised Interview guide for interviews with PHAs unemployed but interested in re-entering work. **Appendix 2C**: Interview guide for interviews with PHAs who have been placed in employment by EA.

## Data Availability

Data sharing is not applicable to this article as no datasets were generated or analysed during the current study.

## References

[1] Haddad N, Robert A, Weeks A, Siu WAC (2019). HIV in Canada—surveillance report, 2017.

[2] Peña Longobardo LM, Oliva-Moreno J (2018). Differences in labour participation between people living with HIV and the general population: results from Spain along the business cycle. PLoS One.

[3] Cooper V, Clatworthy J, Harding R, Whetham J (2017). Measuring quality of life among people living with HIV: a systematic review of reviews. Health Qual Life Outcomes.

[4] Degroote S, Vogelaers D, Vandijck DM (2014). What determines health-related quality of life among people living with HIV: an updated review of the literature. Arch Public Heal.

[5] Dagenais M, Cheng D, Salbach N, Brooks D, O’Brien K (2019). Wireless physical activity monitor use among adults living with HIV: a scoping review. Rehabil Oncol.

[6] Rueda S, Raboud J, Rourke SB, Bekele T, Bayoumi A, Lavis J (2012). Influence of employment and job security on physical and mental health in adults living with HIV: cross-sectional analysis. Open Med.

[7] Public Health Agency of Canada (PHAC). What determines health?. 2011 [cited 2017 Apr 10]. Available from: http://www.phac-aspc.gc.ca/ph-sp/determinants/index-eng.php.

[8] Benach J, Muntaner C (2007). Precarious employment and health: developing a research agenda. J Epidemiol Community Health.

[9] Hacioglu Yildirim M, Alantar Z, Yildirim EA (2014). The relationship between working status and symptoms, quality of life and self-esteem in patients with schizophrenia in Turkey. Int J Soc Psychiatry.

[10] Skarpaas LS, Ramvi E, Løvereide L, Aas RW (2016). Maximizing work integration in job placement of individuals facing mental health problems: supervisor experiences. Work.

[11] Deuchar A. All dressed up with nowhere to go: Transitions to (un)employment for lower middle class young men. Econ Polit Wkly. 2014;(17):104–11. Available from: https://www.scopus.com/inward/record.uri?eid=2-s2.0-84898998255&partnerID=40&md5=54735e8a341c2b2309cd309d6175d3e6.

[12] Kawachi I, Subramanian SV, Almeida-Filho N (2002). A glossary for health inequalities. J Epidemiol Community Health.

[13] Rueda S, Raboud J, Mustard C, Bayoumi A, Lavis JN, Rourke SB (2011). Employment status is associated with both physical and mental health quality of life in people living with HIV. AIDS Care.

[14] Hergenrather KC, Rhodes SD, Clark G (2005). The employment perspectives study: identifying factors influencing the job-seeking behavior of persons living with HIV/AIDS. AIDS Educ Prev.

[15] Ferrier SE, Lavis JN. With health comes work? People living with HIV/AIDS consider returning to work. AIDS Care. 2003;15(3):423–35. 10.1080/0954012031000105478 12828149

[16] Arns PG, Martin DJ, Chernoff RA (2004). Psychosocial needs of HIV-positive individuals seeking workforce re-entry. AIDS Care - Psychol Socio-Medical Asp AIDS/HIV.

[17] Ontario HIV Treatment Network. ECHO employment change and health outcomes study — final report. 2016. Available from: http://www.ohtn.on.ca/wp-content/uploads/2017/02/echo-report.pdf.

[18] Statistics Canada. Labour force survey. 2017. Available from: https://www150.statcan.gc.ca/n1/daily-quotidien/170106/dq170106a-eng.htm.

[19] Brundage V, & Cunningham E. Unemployment holds steady for much of 2016 but edges down in the fourth quarter. US Bureau of Labor Statistics. 2017; Available from: https://www.bls.gov/opub/mlr/2017/article/unemployment-holds-steady-for-much-of-2016-but-edges-down-in-fourth-quarter.htm#:~:text=The number of unemployed and,lower than a year earlier.

[20] Plecher H (2020). Unemployment rate in France 2019.

[21] Blalock AC, McDaniel JS, Farber EW (2002). Effect of employment on quality of life and psychological functioning in patients with HIV/AIDS. Psychosomatics..

[22] Conyers LM, Boomer KB. Validating the client-focused considering work model for people living with HIV and quantifying places of change of commitment to work. Disabil Rehabil. 2017;39(11):1–10. 10.1080/09638288.2016.1180433 27628307

[23] Wagener M, Opstal SV, Miedema H, Gorp EV, Roelofs PD (2017). Work-related stigma and disclosure: a daily challenge for people living with HIV. Work..

[24] Kordovski VM, Woods SP, Verduzco M, Beltran J (2017). The effects of aging and HIV disease on employment status and functioning. Rehabil Psychol.

[25] Wohl DA, Kuwahara RK, Javadi K, Kirby C, Rosen DL, Napravnik S (2017). Financial barriers and lapses in treatment and care of HIV-infected adults in a southern state in the United States. AIDS Patient Care STDs.

[26] Ware D, Rueda S, Plankey M, Surkan P, Okafor CN, Teplin L (2020). The longitudinal impact of employment, retirement and disability status on depressive symptoms among men living with HIV in the Multicenter AIDS Cohort Study. PLoS One.

[27] Stapleton, J, & Tweddle A. Navigating the maze: improving coordination and integration of disability income and employment policies and programs for people living with HIV/AIDS- a discussion paper. 2008. Available from: http://www.realizecanada.org/wp-content/uploads/NavigatingtheMazeFinal.pdf.

[28] Wilton J, Liu J, Sullivan A, et al. New HIV diagnoses in Ontario: Preliminary Update, 2016. Toronto: Ontario HIV Epidemiology and Surveillance Initiative; 2017.

[29] Husbands W. Working positive: a needs assessment of employment action for PHAs. AIDS Committee of Toronto; 2003.

[30] Guest G, Bunce A, Johnson L (2006). How many interviews are enough?: an experiment with data saturation and variability. Field Methods.

[31] Braun V, Clarke V (2006). Using thematic analysis in psychology. Qual Res Psychol.

[32] McArt D (2014). Mental health and barriers to employment. Probat J.

[33] Neumark D, Burn I & BP. Is it harder for older workers to find jobs? New and improved evidence from a field experiment. Natl Bur Econ Res . 2019;127(2):922–970.

[34] Caltaux D (2003). Internalized stigma: a barrier to employment for people with mental illness. Br J Ther Rehabil.

[35] Preibisch K, Hennebry J (2011). Temporary migration, chronic effects: the health of international migrant workers in Canda. Cmaj..

[36] Chambers LA, Rueda S, Baker DN, Wilson MG, Deutsch R, Raeifar E, Rourke SBTSRT (2015). Stigma, HIV and health: a qualitative synthesis. BMC Public Heal.

[37] Rueda S, Mitra S, Chen S, Gogolishvili D, Globerman J, Chambers L, Wilson M, Logie CH, Shi Q, Morassaei SRS (2016). Examining the associations between HIV-related stigma and health outcomes in people living with HIV/AIDS: a series of meta-analyses. BMJ..

[38] Rice WS, Crockett KB, Mugavero MJ, Raper JL, Atkins GC, Turan B (2017). Association between internalized HIV-related stigma and HIV care visit adherence. J Acquir Immune Defic Syndr.

[39] Earnshaw VA, Smith LR, Chaudoir SR, Amico KR, Copenhaver MM (2013). HIV stigma mechanisms and well-being among PLWH: a test of the HIV stigma framework. AIDS Behav.

[40] Liu Y, Canada K, Shi K, Corrigan P. HIV-related stigma acting as predictors of unemployment of people living with HIV / AIDS. 2017;0121(September). 10.1080/09540121.2011.596512 21777074

[41] Martin DJ, Brooks RA, Ortiz DJ, Veniegas RC (2003). Perceived employment barriers and their relation to workforce-entry intent among people with HIV/AIDS. J Occup Health Psychol.

[42] Barrington C, Acevedo R, Donastorg Y, Perez M, Kerrigan D (2017). ‘HIV and work dont go together’: employment as a social determinant of HIV outcomes among men who have sex with men and transgender women in the Dominican Republic. Glob Public Heal.

[43] Bing EG, Burnam A, Longshore D, Fleishman JA, Sherbourne CD, London AS, Turner BJ, Eggan F, Beckman R, Vitiello B, Morton SC, Orlando M, Bozzette SA, Ortiz-Barron LSM (2001). Psychiatric disorders and drug use among human immunodeficienty virus-infected adults. JAMA Psychiatry.

[44] Canada needs universal pharmacare. Lancet. 2019;394(10207):1388. 10.1016/S0140-6736(19)32324-4 31631840

[45] Government of Canada. Summary: Estimates of HIV incidence, prevalence and Canada’s progress on meeting the 90–90-90 HIV targets, 2016. 2018. Available from: https://www.canada.ca/content/dam/phac-aspc/documents/services/publications/diseases-conditions/summary-estimates-hiv-incidence-prevalence-canadas-progress-90-90-90/pub-eng.pdf.

[46] Stringer KL, Turan B, McCormick L, Durojaiye M, Nyblade L, Kempf MC, Lichtenstein BTJ (2017). HIV-related stigma among healthcare providers in the deep south. AIDS Behav.

