# Correction to: A qualitative study of barriers to employment experienced by people living with HIV in Toronto and Ottawa

**DOI:** 10.1186/s12939-021-01434-1

**Published:** 2021-04-07

**Authors:** Melissa Perri, Amy Craig-Neil, Mark Gaspar, Charlotte Hunter, Claire Kendall, Ower Alexander, Andrew D. Pinto

**Affiliations:** 1 Upstream Lab, MAP/Centre for Urban Health Solutions, Unity Health Toronto, Li Ka Shing Knowledge Institute, 30 Bond St, Toronto, Ontario M5B 1W8 Canada; 2 Dalla Lana School of Public Health, University of Toronto, 155 College St, Toronto, Ontario M5T 3M7 Canada; 3 Casey House, 119 Isabella St, Toronto, Ontario M4Y 1P2 Canada; 4 Department of Family and Community Medicine, St. Michael’s Hospital, 500 University Ave, Toronto, Ontario M5G 1V7 Canada; 5 Department of Family Medicine, University of Ottawa, 600 Peter Morand Crescent Suite 201, Ottawa, Ontario K1G 5Z3 Canada; 6 C.T. Lamont Primary Health Care Research Centre, Bruyère Research Institute, 85 Primrose Ave, Ottawa, Ontario K1R 6M1 Canada; 7 Institut du Savoir Montfort, Montfort Hospital, 713 Montreal Rd, Ottawa, Ontario K1K OT2 Canada; 8 Ottawa Hospital Research Institute, 501 Smyth Box 511, Ottawa, Ontario K1H 8L6 Canada; 9 Li Ka Shing Knowledge Institute, 209 Victoria St, Toronto, Ontario M5B 1T8 Canada; 10 University of Toronto Practice-Based Research Network, 500 University Avenue, Toronto, Ontario K1H 8L6 Canada

**Correction to: Int J Equity Health 20, 36 (2021)**

**DOI: 10.1186/s12939-020-01356-4**

Following publication of the original article [1], the authors identified that one of the authors’ names was mentioned incorrectly. It should be as per below:

First name - Amy and last name Craig-Neil.
