# Supplementary material for: A qualitative study of barriers to employment experienced by people living with HIV in Toronto and Ottawa
Source: Int J Equity Health. 2021 Jan 14;20:36. doi: 10.1186/s12939-020-01356-4 (PMC7807879; doi:10.1186/s12939-020-01356-4)
Supplement: Supplementary file 1 — Additional file 1. Recruitment Posters for PLWHs Employed and Unemployed. [file 12939_2020_1356_MOESM1_ESM.zip › Appendix_1A_-Recruitment_poster.pdf]

INVITATION

To Participate in a Research Interview

STUDY TITLE: Work, Health and Living with HIV

WHAT IS IT ABOUT?

We are studying the barriers faced by people living with HIV hoping to re-enter the workforce and the supports that they need.

WHO ARE WE LOOKING FOR?

Patients living with HIV who are not currently working but interested in re-entering the workforce.

HOW LONG WILL IT TAKE?

The interview will last between 30 and 60 minutes.

WILL I BE COMPENSATED?

All participants will receive $20.

DO I HAVE TO PARTICIPATE?

No! Participation in the study is completely **voluntary.**

IS IT CONFIDENTIAL?

Yes! No one outside the research team will know if you participate. All information will be securely stored.

WANT TO PARTICIPATE?

Contact Amy Craig-Neil at 416-864-6060 Ext. 76148 or craigneila@smh.ca

*Disability accommodation can be provided.

This study has been approved by the Ottawa Health Science Network, Bruyere and Unity Health Research Ethics Boards SMH REB#18-274 07/12/2018. Contact: 416-864-6060 Ext. 2557.
