# Supplementary material for: A qualitative study of barriers to employment experienced by people living with HIV in Toronto and Ottawa
Source: Int J Equity Health. 2021 Jan 14;20:36. doi: 10.1186/s12939-020-01356-4 (PMC7807879; doi:10.1186/s12939-020-01356-4)
Supplement: Supplementary file 1 — Additional file 1. Recruitment Posters for PLWHs Employed and Unemployed. [file 12939_2020_1356_MOESM1_ESM.zip › Appendix_1B_-_Recruitment_poster.pdf]

INVITATION

To Participate in a Research Interview

STUDY TITLE: Work, Health and Living with HIV

WHAT IS IT ABOUT?

We would like to learn about the experiences of people who have been through the Employment ACTion program.

WHO ARE WE LOOKING FOR?

People who have been successfully placed into work by Employment ACTion.

HOW LONG WILL IT TAKE?

The interview will last between 30 and 60 minutes.

WILL I BE COMPENSATED?

All participants will receive $20.

DO I HAVE TO PARTICIPATE?

No! Participation in the study is completely **voluntary.**

IS IT CONFIDENTIAL?

Yes! No one outside the research team will know if you participate. All information will be securely stored.

WANT TO PARTICIPATE?

Contact Amy Craig-Neil at 416-864-6060 Ext. 76148 or craigneila@smh.ca

*Disability accommodation can be provided.

This study has been approved by the Ottawa Health Science Network, Bruyere and Unity Health Research Ethics Boards SMH REB#18-274 07/12/2018. Contact: 416-864-6060 Ext. 2557.
