# Supplementary material for: A qualitative study of barriers to employment experienced by people living with HIV in Toronto and Ottawa
Source: Int J Equity Health. 2021 Jan 14;20:36. doi: 10.1186/s12939-020-01356-4 (PMC7807879; doi:10.1186/s12939-020-01356-4)
Supplement: Supplementary file 2 — Additional file 2: Appendix 2A: Interview guide for interviews with PHAs unemployed but interested in re-entering work. Appendix 2B: Revised Interview guide for interviews with PHAs unemployed but interested in re-entering work. Appendix 2C: Interview guide for interviews with PHAs who have been placed in employment by EA. [file 12939_2020_1356_MOESM2_ESM.zip › Appendix_2A_-_Interview_Guide.docx]

**Appendix 2A: Interview guide for interviews with PHAs unemployed but interested in re-entering work**

Purpose: to understand your previous work experiences (and stopping work) as well as reasons, barriers and facilitators to re-entering into work.

Preamble:

1. What motivated you to come and do this interview?
2. I know that we just completed the survey around your health but can you quickly describe your overall health as well as any HIV related challenges that you have faced.

**Part 1: Personal experience with work, unemployment and re-entry**

1. Tell me a bit about your previous training, education and work history.
   1. When did you last work? How long ago? What sort of job?
   2. Why did you stop working there? What was that experience like?
2. Describe the experience being unemployed
   1. Stigma? By who?
   2. Do you volunteer currently or engage in unpaid work (e.g. informal assistance to others)? Did you learn any new skills or doing any further education or training?
   3. Past history of unemployment?
3. Can you describe how living with HIV has affected:
   1. Your ability to work
   2. Your relationship to work (i.e. disclosing status, taking time off…)
   3. Were there other impediments to work?
4. I understand you’ve been thinking about re-entering work. Can you tell me about that?
   1. Why?
   2. What are you looking for?
   3. You haven’t worked in _____ years, how does that affect your search/how you feel about re-entering work?
   4. Do you have any concerns about pursuing a career now?
      1. Disclosing status
      2. Sick days
      3. Losing drug benefits
5. Do you face barriers to re-entering the workforce? If yes, what are they?
6. What do you think would help you get a job?
   1. Supports?
   2. Info?
   3. Skills/training?
7. Have you accessed any resources/agencies/services you know of to assist with re-entry?
   1. Why or why not?
   2. How did you hear about them?
8. Is there any role that the health care team/your physician play or could play in helping you with employment?
   1. Have you discussed this with them?
   2. Have they encouraged/discouraged you to work?
   3. Relationship with HC team

**Part 2: Thoughts on intervention study**

We are thinking about seeking funds to carry out a study of helping PHA re-enter work, bringing together health providers with specialists in employment.

1. Would this be of interest to you?
2. Why?
3. Describe how you imagine this sort of program. What would they provide?
4. Where and how would we best engage participants?
   1. Health setting? Community HIV/AIDS agency e.g. PWA, ACT? Community agency not associated with HIV/AIDS?
5. Would you want your doctor to be involved in this process?
   1. Why?
   2. Benefits/drawbacks?
6. Would you be comfortable if your data was collected and you were contacted in follow-up from researchers studying the intervention?

The purpose of this study is to understand barriers for those living with HIV in re-entering work, what supports may help this transition and how the healthcare team can assist in this process. Knowing that, do you feel like there is anything missed in this interview? Or anything else that you would like to add?

Thanks.
