# Supplementary material for: A qualitative study of barriers to employment experienced by people living with HIV in Toronto and Ottawa
Source: Int J Equity Health. 2021 Jan 14;20:36. doi: 10.1186/s12939-020-01356-4 (PMC7807879; doi:10.1186/s12939-020-01356-4)
Supplement: Supplementary file 2 — Additional file 2: Appendix 2A: Interview guide for interviews with PHAs unemployed but interested in re-entering work. Appendix 2B: Revised Interview guide for interviews with PHAs unemployed but interested in re-entering work. Appendix 2C: Interview guide for interviews with PHAs who have been placed in employment by EA. [file 12939_2020_1356_MOESM2_ESM.zip › Appendix_2C_-_Interview_guide_for_PHAs.docx]

**Appendix 2C**: **Interview guide for interviews with PHAs who have been placed in employment by EA**

**Part 1: Intro and personal experience with work, unemployment and re-entry**

1. What motivated you to come and do this interview?
2. I know that we just completed the survey around your health but can you quickly describe your overall health as well as any HIV related challenges that you have faced.
3. Tell me a bit about your previous training, education and work history (prior to working with Employment ACTion).
   1. When did you last work? How long ago? What sort of job?
   2. Why did you stop working there? What was that experience like?
4. Describe the experience being unemployed
   1. Stigma? By who?
   2. Do you volunteer currently or engage in unpaid work (e.g. informal assistance to others)? Did you learn any new skills or doing any further education or training?
   3. Past history of unemployment?
5. Can you describe how living with HIV has affected:
   1. Your ability to work
   2. Your relationship to work (i.e. disclosing status, taking time off…)
   3. Were there other impediments to work?
6. Did you experience barriers prior to re-entering the workforce?

**Part 2: Experience with Employment ACTion**

1. How did you find out about/get connected to Employment ACTion?
2. Please describe your experience with Employment ACTion.
   1. How did they help?
   2. What supports did they provide you with?
   3. What do you think was the most important thing that they provided in your transition back into the workforce?
   4. How do you think their service could have been improved?
3. Can you please describe your current work?
   1. Do you face barriers in maintaining your job?
   2. What supports do you require to maintain your job?
   3. Are you happy with your current job? Did it align with the employment goals that you had when you started with EA?
4. Has your health care provider been involved in the process?
   1. In what way?

**Part 3: Thoughts on intervention study**

We are thinking about seeking funds to carry out a study of helping PHA re-enter work, bringing together health providers with specialists in employment.

1. Do you think this would be useful for other people living with HIV to re-enter work?
   1. The benefits of having health providers involved?
   2. The drawbacks of health providers involved?
2. Where would it be best to engage people?
   1. Health setting?
   2. Community HIV/AIDS agency e.g. PWA, ACT
   3. Community agency not associated with HIV/AIDS
3. What supports do you think would be particularly useful in a program like this?
4. Would you want your doctor to be involved in this process?
   1. Why?
   2. Benefits/drawbacks?
5. Would you be comfortable if your data was collected and you were contacted in follow-up from researchers studying the intervention?

The purpose of this study is to understand barriers for those living with HIV in re-entering work, what supports may help this transition and how the healthcare team can assist in this process as well as your experience working with Employment ACTion. Knowing that, do you feel like there is anything missed in this interview? Or anything else that you would like to add?
